# Schizophrenia and Sleep Disorders: An Introduction

**DOI:** 10.3390/medicines5030094

**Published:** 2018-08-30

**Authors:** Maurits van den Noort, Peggy Bosch

**Affiliations:** 1 Research Group of Pain and Neuroscience, Kyung Hee University, Seoul 130-701, Korea; 2 Brussels Institute for Applied Linguistics, Vrije Universiteit Brussel, 1050 Brussels, Belgium; 3 Psychiatric Research Group, LVR-Klinik Bedburg-Hau, 47511 Bedburg-Hau, Germany; 4 Donders Institute for Brain, Cognition and Behaviour, Radboud University, 6525 Nijmegen, The Netherlands

This editorial is an introduction to the special issue ‘Schizophrenia and Sleep Disorders’. It provides a brief overview of the research field, discusses the four main studies of the special issue, and gives some important directions for future research.

Schizophrenia is characterized by the so-called positive symptoms, negative symptoms, and cognitive symptoms [1] (see Figure 1). Contrary to what might be thought, the positive symptoms are not positive for the patient; rather, the name solely refers to symptoms that are “added to” or visible in patients with schizophrenia, but not in healthy individuals. Here, for the typical patient with schizophrenia, one can think of auditory-, visual-, olfactory-, tactile-, or gustatory- hallucinations and delusions, etc. [2]. Examples of negative symptoms (symptoms that are visible in healthy individuals, but not in patients with schizophrenia) are, for instance, the flat affect, inability to experience pleasure, emotional withdrawal, active social avoidance, lack of motivation, etc. [3]. Problems in executive functioning, including problems with working memory [4], focused and sustained attention, and problem solving, deficits in response initiation and suppression, etc. [5], are examples of cognitive symptoms. Schizophrenia is a severe psychiatric disorder and has a large impact on the patient’s life. Most patients suffer from the disorder for a long time, even a lifetime, and the costs for society are high [6]. The severity of the disorder manifests itself in a high suicide rate, with about 10% of the patients suffering from schizophrenia committing suicide [5].

Schizophrenia is relatively common. Its worldwide prevalence is estimated to be around 0.5% [7], and its occurrence appears to be related to genetics [8]. For instance, a number of common genetic variants, rare copy-number variants, and ultra-rare gene-disrupting mutations have been found to play a role in schizophrenia [9]. Less familiar to the general public is the fact that most patients with schizophrenia also suffer from sleep problems [10] (see Figure 1). Common disturbances reported in clinical studies are reduced sleep efficiency, reduced total sleep time, and increased sleep latency [11]. Even more surprisingly is the fact that those sleep disturbances in patients with schizophrenia are frequently not accounted for in daily clinical practice [11].

The aim of this special issue will be to provide an overview of the specific sleep disturbances experienced by patients with schizophrenia and the negative effects of those disturbances on cognition; moreover, the different kinds and combinations of treatment techniques available to date will be discussed, with a focus on the following question: How can one develop the best treatment for sleep disorders in patients with schizophrenia, sufficiently taking into account the different stages of the disorder and the individual differences between the patients?

The first study by Assimakopoulos and colleagues [12] in our special issue focuses on the topic of genetic variations in patients suffering from schizophrenia and co-morbid sleep disorders. From previous research, genetics is known to play an important role in schizophrenia, indicating that some individuals are genetically more at risk to develop schizophrenia later in life than others [8]. The study by Assimakopoulos et al. [12] is unique because it taps not only into genetics but also into the co-morbid sleep disorders of patients with schizophrenia. In their study, the authors searched the PubMed, ScienceDirect, and GoogleScholar databases and followed the guidelines of the Preferred Reporting Items for Systematic Reviews and Meta-analysis (PRISMA) protocol. In total, 11 eligible studies were found. The results revealed that certain genetic variations were indeed found to be associated with schizophrenia-related sleep disorders. For instance, antipsychotic-induced restless legs syndrome was linked to polymorphisms located on the CLOCK, BTBD9, GNB3, and TH genes. Moreover, clozapine-induced somnolence was correlated with polymorphisms of the HNMT gene. In addition, insomnia was associated with variants of the MTNR1 gene. The authors conclude that significant genetic associations exist between schizophrenia and co-morbid sleep disorders, implicating the circadian system, dopamine and histamine metabolism, and signal transduction pathways [12].

The second article by Bosch and colleagues [13] focuses on the pharmacological treatment of long-term patients with schizophrenia, which is still the cornerstone of disease management. However, how pharmacological treatment affects sleep and whether gender differences exist remain unclear, so these questions were the main issues that were investigated. To that end, the numbers of antipsychotics, sleep medications, antidepressants, and anxiolytics used by 46 long-term outpatients with schizophrenia were analyzed. In addition, the scores of the patients on the Pittsburgh Sleep Quality Index (PSQI) [14] and the Epworth Sleepiness Scale (ESS) [15] were analyzed. The results of the study revealed a large individual variability; some patients received no psychiatric drugs at all whereas other patients received up to eight different psychiatric medications at a time. Moreover, the patients were found to have high scores on the PSQI, but not on the ESS. This means that although the patients with schizophrenia report problems with sleep, they do not feel sleepy during the day. A significant positive correlation between the use of antipsychotics and the ESS score, but not the PSQI score, was found. The statistical analyses of the data revealed no gender differences in the pharmacological treatment of long-term patients with schizophrenia. The authors conclude that a large variability exists in the pharmacological treatment of such patients. Moreover, to date, not enough attention has been paid to the treatment of schizophrenic patients’ sleep problems and to the gender differences in the pharmacological treatment of those patients. Finally, the authors stress the point that a need exists for more and larger replication studies in order to verify the present study findings and in order to recommend any changes in the drug treatment of male and female patients with long-term schizophrenia.

The third study by Stummer and colleagues [16] goes deeper into the pharmacological treatment of sleep disorders in patients with schizophrenia. The authors conducted a literature search through OVID and PubMed to find relevant publications on pharmacotherapy options to treat insomnia in patients with schizophrenia. The results of that review show that only a few evidence-based options are available to treat insomnia in patients with schizophrenia, including selecting a more sedating second-generation antipsychotic, such as paliperidone, or adding melatonin or eszopiclone. However, the authors stress the important need for further randomized controlled trials to verify these findings.

The fourth study by Van den Noort and colleagues [17] taps the issue that, in addition to Western medicine, the use of other methods, such as acupuncture, to treat the positive, negative, and cognitive symptoms of patients with schizophrenia is on the rise. A systematic review that included all studies involving the use of standard acupuncture in the treatment of patients with schizophrenia was conducted. The authors searched the Medline, ScienceDirect, Cochrane Library, Scopus, and ERIC databases. Moreover, the study was conducted in accordance with the PRISMA protocol. Twenty-six studies fulfilled all selection criteria. In total, 1181 patients with schizophrenia were treated with acupuncture. The results showed that most studies found only small beneficial effects of acupuncture in the treatment of the positive, negative, and cognitive symptoms. However, larger beneficial effects were found regarding the treatment of the accompanying sleep disorders in patients with schizophrenia, leading the authors to propose the “Acupuncture–Dopamine–Sleep” (ADS) hypothesis. It states that “acupuncture is able to affect the disturbed and hyperactive dopamine system in patients with schizophrenia, bringing it back into balance, and as a result, the patients start to feel and function better, without necessarily getting rid of all their clinical symptoms” [17]. Finally, the authors stress the point that the results found in their review study need to be confirmed by future large, randomized, controlled trials that to date are, unfortunately, to a large extent missing.

In future research, if a better understanding of genetic variations in patients with schizophrenia and co-morbid sleep disorders is to be achieved, more studies on the circadian system, dopamine and histamine metabolism, and signal transduction pathways in such patients are required. Moreover, future pharmacological studies should focus more on the patients’ sleep problems. In addition, individual and gender differences need to be taken into account if a more optimal pharmacological treatment is to be found. With respect to the usefulness of acupuncture as an add-on treatment for patients with schizophrenia and co-morbid sleep disorders, the ADS hypothesis seems to be a promising working hypothesis to focus on in future acupuncture research. (For an overview of the main topics for future research, see Figure 2.)

Taking all of the above together, one can conclude that to date, an optimal treatment for patients with schizophrenia and co-morbid sleep disorders may still be a long way off. Much research remains to be done before the relation between genetics and clinical symptoms is fully understood and, as a result, before good individual-based (pharmacological) treatments can be offered. One must remain skeptical about, and critically assess, the limited treatment successes obtained so far. A hope of the authors is that important progress will soon be made in the treatment of patients with schizophrenia and co-morbid sleep disorders so that the great suffering of those patients and their families and friends can be reduced. The authors hope that the subjects of the present special issue will stimulate discussion and encourage research on the way to achieve this goal.

## Figures and Tables

**Figure 1:**
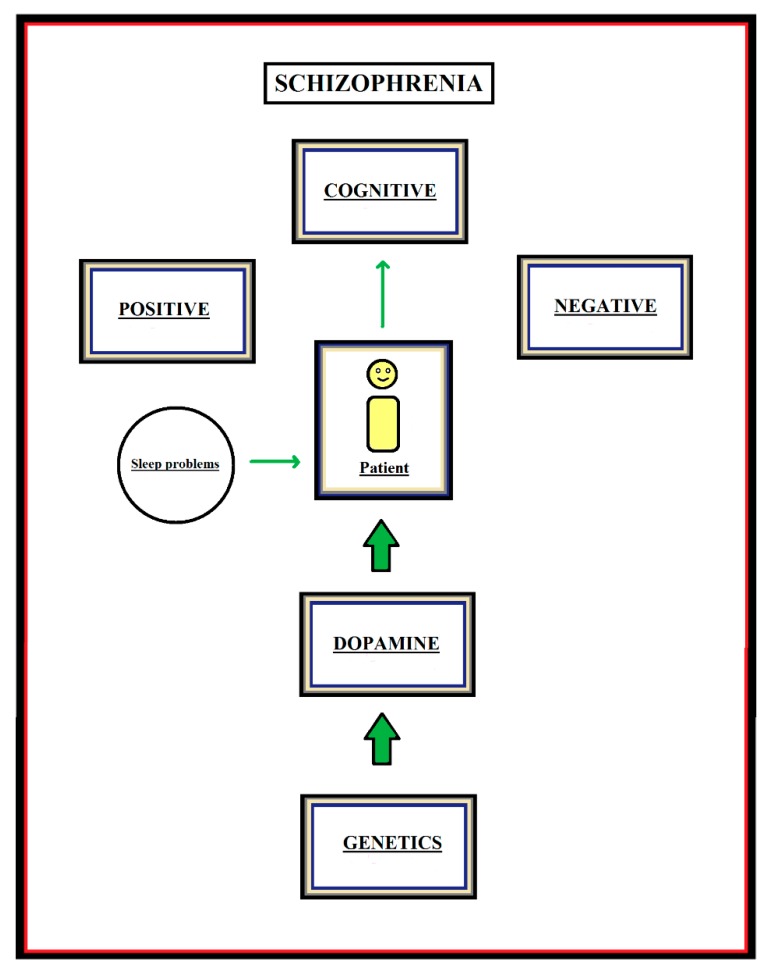
Overview of the clinical symptoms of patients with schizophrenia and the key factors that are the focus of the present special issue and are suggested to influence those symptoms.

**Figure 2:**
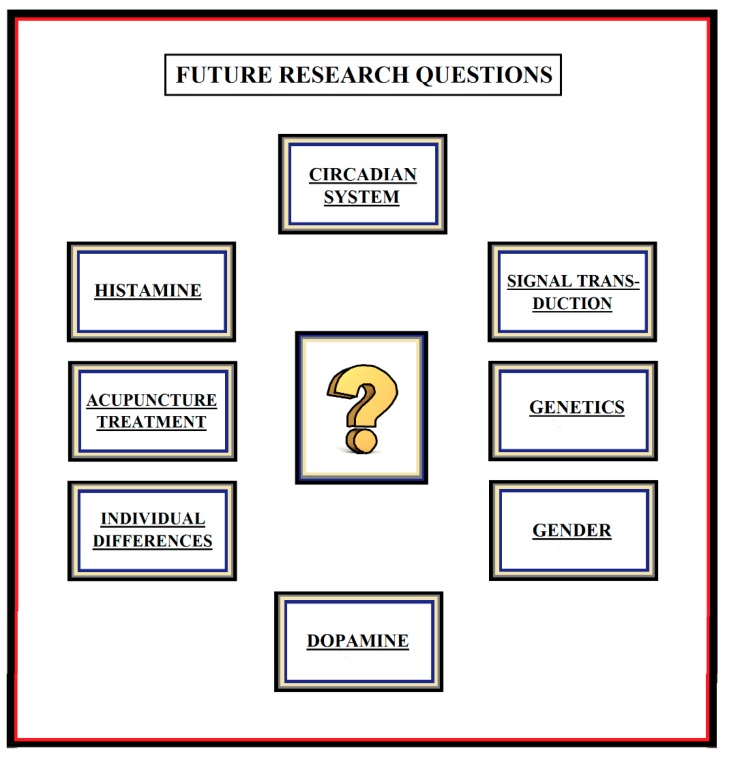
Important research questions that need to be further investigated in future research.

## References

[1] Carbon M., Correll C.U. (2014). Thinking and acting beyond the positive: The role of the cognitive and negative symptoms in schizophrenia. CNS. Spectr..

[2] Teeple R.C., Caplan J.P., Stern T.A. (2009). Visual hallucinations: Differential diagnosis and treatment. Prim. Care Companion J. Clin. Psychiatry.

[3] Foussias G., Remington G. (2010). Negative symptoms in schizophrenia: Avolition and occam’s razor. Schizophr. Bull..

[4] Van den Noort M., Struys E., Perriard B., Staudte H., Yeo S., Lim S., Bosch P. (2016). Schizophrenia and depression: The relation between sleep quality and working memory. Asian J. Psychiatr..

[5] Orellana G., Slachevsky A. (2013). Executive functioning in schizophrenia. Front. Psychiatry.

[6] Evensen S., Wisløff T., Lystad J.U., Bull H., Ueland T., Falkum E. (2016). Prevalence, employment rate, and cost of schizophrenia in a high-income welfare society: A population-based study using comprehensive health and welfare registers. Schizophr. Bull..

[7] Messias E.L., Chen C.Y., Eaton W.W. (2007). Epidemiology of schizophrenia: Review of findings and myths. Psychiatr. Clin. N. Am..

[8] Delisi L.E. (2009). Searching for the true genetic vulnerability for schizophrenia. Genome Med..

[9] Stokowy T., Polushina T., Sønderby I.E., Karlsson R., Giddaluru S., Le Hellard S., Bergen S.E., Sullivan P.F., Andreassen O.A., Djurovic S. (2018). Replication of association to lipid biosynthesis genes. Sci. Rep..

[10] Cohrs S. (2008). Sleep disturbances in patients with schizophrenia: Impact and effect of antipsychotics. CNS Drugs.

[11] Van den Noort M., Staudte H., Perriard B., Yeo S., Lim S., Bosch P. (2016). Schizophrenia and comorbid sleep disorders. Neuroimmunol. Neuroinflamm..

[12] Assimakopoulos K., Karaivazoglou K., Skokou M., Kalogeropoulou M., Kolios P., Gourzis P., Patrinos G.P., Tsermpini E.E. (2018). Genetic variations associated with sleep disorders in patients with schizophrenia: A systematic review. Medicines.

[13] Bosch P., Lim S., Staudte H., Yeo S., Lee S.H., Barisch P., Perriard B., Van den Noort M. (2018). Pharmacological treatment for long-term patients with schizophrenia and its effects on sleep in daily clinical practice: A pilot study. Medicines.

[14] Buysse D.J., Reynolds C.F., Monk T.H., Berman S.R., Kupfer D.J. (1989). The Pittsburgh Sleep Quality Index: A new instrument for psychiatric practice and research. Psychiatry Res..

[15] Johns M.W. (1991). A new method for measuring daytime sleepiness: The Epworth sleepiness scale. Sleep.

[16] Stummer L., Markovic M., Maroney M. (2018). Pharmacologic treatment options for insomnia in patients with schizophrenia. Medicines.

[17] Van den Noort M., Yeo S., Lim S., Lee S.H., Staudte H., Bosch P. (2018). Acupuncture as add-on treatment of the positive, negative, and cognitive symptoms of patients with schizophrenia: A systematic review. Medicines.

